# Impacts of volumetric vs. anatomical accuracies in GTV segmentation for nasopharyngeal carcinoma: a consensus-guideline-based study

**DOI:** 10.3389/fmed.2026.1941973

**Published:** 2026-09-08

**Authors:** Jiayang Lu, Yangkang Li, Yuanxiang Yu, Longjia Guo, Yu Shao, Cuidai Zhang, Zhixiong Lin, Yong Gan

**Affiliations:** 1 Department of Radiotherapy, Cancer Hospital of Shantou University Medical College, Shantou University, Shantou, China; 2 Department of Radiology, Cancer Hospital of Shantou University Medical College, Shantou University, Shantou, China

**Keywords:** accuracy, consensus guideline, dosimetry, geometry, nasopharyngeal carcinoma, target volume segmentation, tumor control probability

## Abstract

**Background:**

Consensus guidelines for target volume segmentation have been issued for nasopharyngeal carcinoma (NPC) radiotherapy, yet prior studies have largely overlooked anatomical accuracy in this context.

**Methods:**

Sixteen NPC patients were enrolled. GTVnx and prophylactic clinical target volume of primary tumor (CTVp) were independently contoured by three radiation oncologists and one senior radiologist to obtain observer segmentations (OSs) and reference GTVnx. Original CTVp of OS were adjusted according to reference GTVnx. CTVp consistency was evaluated with Dice Similarity Coefficient (DSC), 95th Hausdorff Distance (HD95) and Intraclass Correlation Coefficient (ICC). Impacts of volumetric and anatomical accuracy were evaluated with precision and true positive rate (TPR), dosimetric parameters and tumor control probability (TCP).

**Results:**

Anatomical accuracy was strikingly high (median anatomical TPR: 89.5%–100%; precision: 80%–92%), volumetric accuracy was substantially lower (median volumetric TPR: 63.88%–73.92%; precision: 47.08%–70.29%). Tiny differences were observed in CTVp consistency between original and adjusted CTVp (Median DSC: 0.84 vs. 0.85, Median HD95: 6.53 vs. 6.27 mm, ICC: 0.98 vs. 0.99), which were both higher than that of GTVnx consistency across OSs (Median DSC: 0.63, Median HD95: 8.21 mm, ICC: 0.92). Treatment plans based on OS resulted in decreased D95, EUD and TCP (average decrease of D95, EUD and TCP: 7.38 Gy, 5.24 Gy and 17.13%).

**Conclusions:**

In NPC, primary tumor segmentation inaccuracies are predominantly volumetric rather than anatomical. Low volumetric accuracy may potentially reduce TCP. Under consensus guidelines, high anatomical accuracy in GTVnx segmentation may contribute to consistent CTVp segmentation.

## 1 Introduction

Nasopharyngeal carcinoma (NPC) is a highly prevalent head and neck cancer (HNC) in Southeast Asia, with an annual incidence of 20 per 100,000 population (1). Modern radiotherapy techniques, including intensity-modulated radiation therapy (IMRT) and volumetric modulated arc therapy (VMAT), have become the standard of care. Compared to conventional radiotherapy, IMRT and VMAT enable steep dose gradients around the target volume, achieving highly conformal dose distributions. This allows escalation of tumor dose while sparing adjacent normal tissues, thereby improving the therapeutic ratio by increasing tumor control probability (TCP) and reducing normal tissue complication probability (NTCP) (2, 3).

Highly conformal dose distribution of modern radiotherapy requires oncologists' accurate segmentation of primary tumor and consistent segmentation of clinical target volume (CTV). To improve the accuracy of gross tumor volume (GTV) segmentation, multimodality imaging, educational courses and consensus guidelines have been developed and proved effectiveness (4–6). For CTV segmentation of NPC, international guideline provides detailed recommendations based on available evidence, protocol comparisons and expert consensus (7–9). This guideline has been proved the capability to improve CTV consistency (6, 10) and endorsed by the National Comprehensive Cancer Network (NCCN) (11).

According to the international guideline, prophylactic CTV of primary tumor (CTVp) is based on invaded anatomical structures. Consequently, the consistency of CTVp segmentation between oncologists may be influenced by the underlying accuracy of nasopharyngeal GTV (GTVnx) segmentation. Previous studies evaluating accuracy of GTVnx segmentation have relied predominantly on volumetric similarity metrics, such as Dice Similarity Coefficient (DSC) and the 95th Hausdorff Distance (HD95) et al. These metrics limited the understanding of clinical relevance of GTV segmentation accuracy. First from the point of guideline application, the CTVp is based on invaded anatomical structure more than the volumetric extent of invasion, therefore volumetric evaluation of the GTV alone is insufficient and easily bias the conclusions due to large discrepancies in volume size of different anatomical structures around nasopharynx. Second, volumetric similarity provides no direct information on resultant dosimetry deviations and let alone the potential biological consequences for TCP.

To address these gaps, this study dissected GTVnx contour inaccuracy into volumetric and anatomical components. Specifically, we aimed to: (1) quantify GTVnx segmentation accuracy at both volumetric and anatomical structure levels; (2) evaluate the dosimetric and biological impacts—particularly TCP reduction—resulting from volumetric inaccuracy; and (3) investigate how GTVnx segmentation accuracy affects CTVp consistency within the framework of the international consensus guidelines.

## 2 Methods and materials

### 2.1 Patients selection

Patients were retrospectively selected from the inpatients recording system of our hospital according to following inclusion criteria: (1) Histopathological confirmed keratinizing and non-keratinizing nasopharyngeal carcinoma according to WHO 2003 classification; (2) Stage of T1–4 and N1–3 based on AJCC 9th edition; (3) Age between 18 and 70 years; (4) Received definitive radiotherapy. Exclusion criteria: (1) Presence of non-removable metal dentures; (2) prior head and neck radiotherapy or surgery; (3) Contraindications to contrast-enhanced CT or MRI.

### 2.2 CT and MRI acquisition

All patients underwent contrast-enhanced CT and MRI before radiotherapy. CT-scans were performed with Brilliance CT Big Bore CT scanner (Koninklijke Philips N.V., Amsterdam, Netherlands). The scanning range extended from 2 cm above the superior orbital margin to 2 cm below the inferior edge of the clavicle, with adjustments based on the extent of the lesion. The Slice thickness and spacing were both 3 mm.

MRI was performed on a 3.0 T system (Discovery MR750, GE Medical Systems, Milwaukee, WI, USA), including T1/T2-weighted and fat-suppression sequences.

CT and MRI images were transferred via the DICOM to the treatment planning system (Eclipse Version 16.1, Varian Medical Systems. Eclipse treatment planning system, Palo Alto, CA, USA).

### 2.3 Target volume and dose prescription

**Figure 1:**
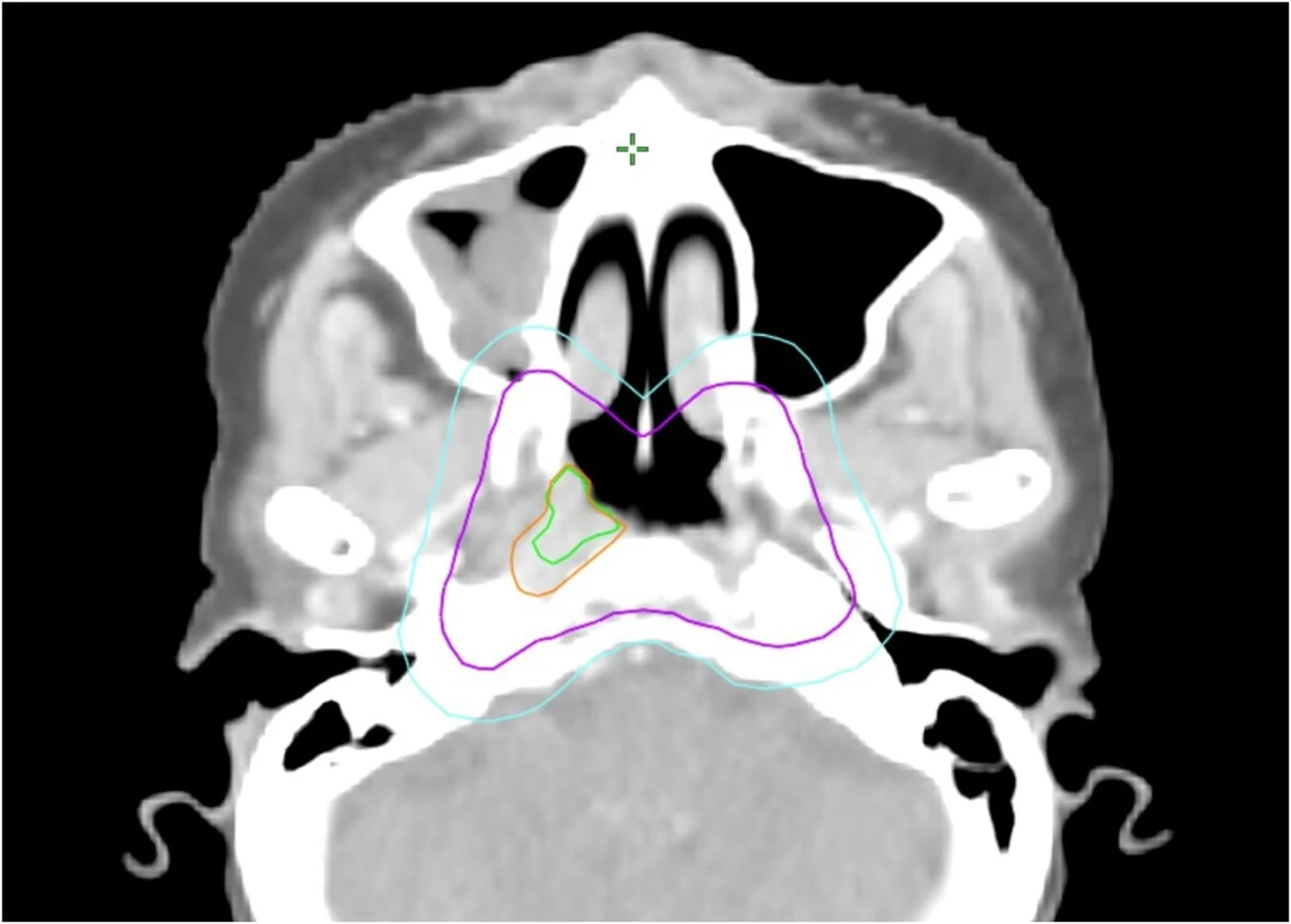
Target volume segmentation for a representative patient. Axial CT image shows one observer's segmentation of GTVnx (GTVnx_OS: brown), reference segmentation of GTVnx (GTVnx_ref: green), CTV (red: original, blue: adjusted) and PTV (light blue). Original CTV and adjusted CTV are perfectly overlapped (purple) due to high anatomical accuracy.

The international consensus guideline for GTV and CTV segmentation (7, 8) was adopted to eliminate the impacts of differing protocols. To eliminate the interaction effects between different dose-level CTVs, the high-dose CTV were excluded, as it is merely a geometric expansion from GTV, therefore only low-dose CTV of primary tumor and nodal regions were evaluated. A uniform 5-mm margin was applied to produce planning target volume (PTV). The prescription dose to PTV of primary tumor, metastatic lymph nodes and low-dose CTV are 70 Gy, 70 Gy and 54 Gy. The detailed definition and prescription dose to different target volumes are shown in Supplementary Table 1. The dose constraints for different organs are shown in Supplementary Table 2. A representative patient for target volume segmentation is shown in Figure 1.

### 2.4 Reference and observer segmentation, original and adjusted CTV

MRI images were rigidly registered to CT images using a control region from the skull base to the caudal edge of the C2 vertebral body to assist GTVnx segmentation.

Reference GTVnx and Gross lymph node volume (GTVln) were segmented by the same senior radiologist of multidisciplinary team in our hospital for all patients and designated as GTVnx_ref and GTVln_ref.

Observer segmentation (OS) of GTVnx, GTVln and CTV were independently performed by three radiation oncologists (each with >10 years of experience) to obtain GTVnx_OS1/2/3, GTVln_OS1/2/3 and original CTV_OS1/2/3.

Each oncologist then adjusted their original CTV based on the GTVnx_ref and GTVln_ref to obtain adjusted CTV_OS1/2/3.

To exclude the impacts of GTVln, a transverse plane 1 cm below the caudal edge of GTVnx_ref (Plane A) was defined. The cranial section of CTV, representing the CTVp, was analyzed with its corresponding planning target volume (PTVp).

Transverse plane at the bottom of the sphenoid sinus (Plane B) was defined to divide GTVnx and CTVp into superior and inferior sections (with suffix: _sup and _inf).

**Figure 2:**
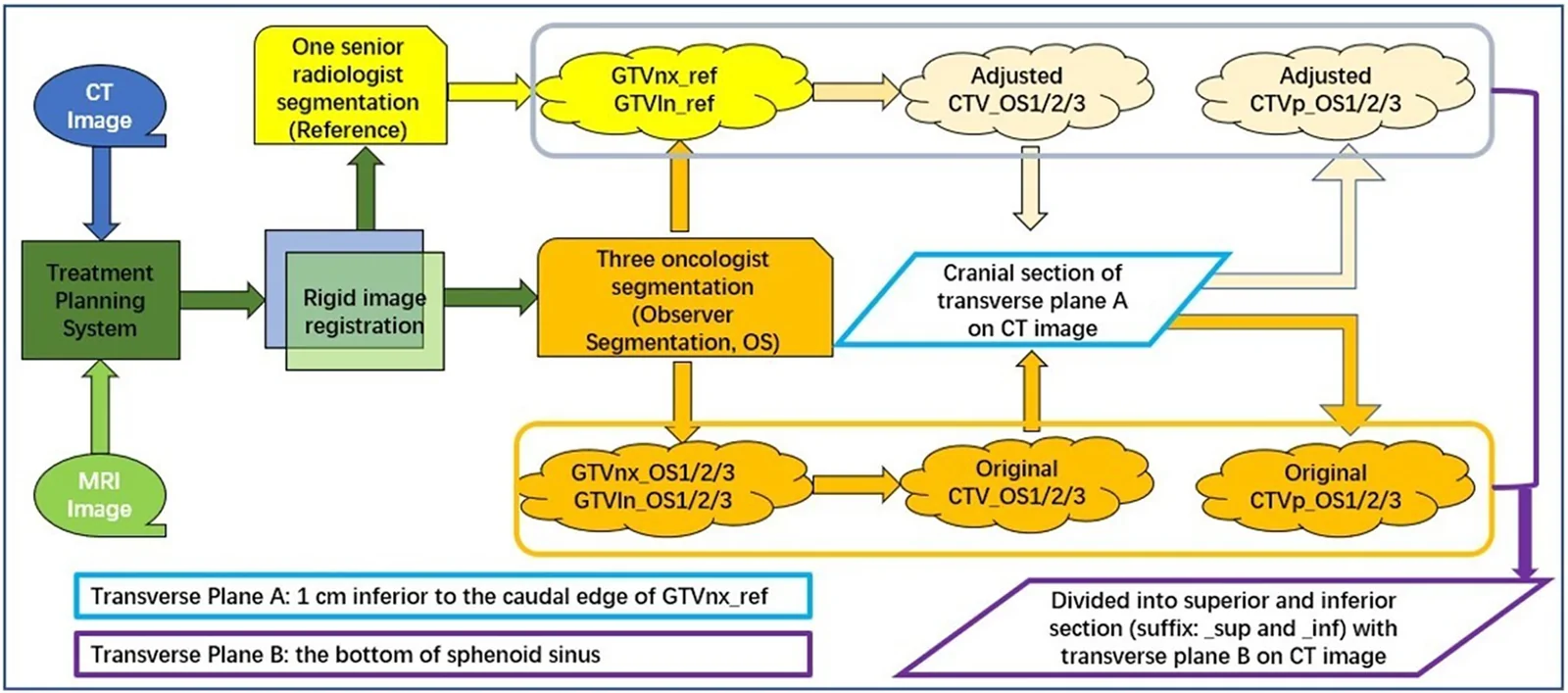
Workflow of target volume segmentation and designation.

The Figure 2 illustrates the segmentation workflow and target volume designation.

### 2.5 Treatment planning

Treatment plans were generated using VMAT technique based on OS. Three coplanar full arcs were employed with 6-MV photon beams delivered by a TrueBeam linear accelerator (Varian Medical Systems, Palo Alto, CA, USA).

The first two arcs used collimator angles between 5°–30° and 330°–350° to enhance multi-leaf collimator (MLC) modulation, the third arc collimator was set at 0°. Dose-limiting ring structures were applied to maintain steep dose gradients.

### 2.6 Volumetric and anatomical accuracy of GTVnx segmentation

The precision and true positive rate (TPR) at volumetric level were calculated with following Formulas 1, 2:

$$\mathrm{Precision}=\frac{\left(\mathrm{Volume}\;\mathrm{of}\;\mathrm{GTVnx}\_\mathrm{OS})\;\cap (\mathrm{Volume}\;\mathrm{of}\;\mathrm{GTVnx}\_\mathrm{ref}\right)}{\mathrm{Volume}\;\mathrm{of}\;\mathrm{GTVnx}\_\mathrm{OS}}\times 100\text{\%}$$ (1)

$$\mathrm{True}\;\mathrm{positive}\;\mathrm{rate}\;(\mathrm{TPR})=\frac{\left(\mathrm{Volume}\;\mathrm{of}\;\mathrm{GTVnx}\_\mathrm{OS})\;\cap (\mathrm{Volume}\;\mathrm{of}\;\mathrm{GTVnx}\_\mathrm{ref}\right)}{\mathrm{Volume}\;\mathrm{of}\;\mathrm{GTVnx}\_\mathrm{ref}}\times 100\text{\%}$$ (2)

The precision and TPR at anatomical level were calculated with following Formulas 3, 4:

$$\mathrm{Precision}=\frac{\mathrm{Number}\;\mathrm{of}\;(\mathrm{invaded}\;\mathrm{structure}\;\mathrm{of}\;\mathrm{GTVnx}\_\mathrm{OS}\;\cap \;\mathrm{invaded}\;\;\mathrm{structure}\;\mathrm{of}\;\mathrm{GTVnx}\_\mathrm{ref})}{\mathrm{Number}\;\mathrm{of}\;\mathrm{invaded}\;\mathrm{structure}\;\mathrm{of}\;\mathrm{GTVnx}\_\mathrm{OS}}\times 100\text{\%}$$ (3)

$$\mathrm{TPR}=\frac{\mathrm{Number}\;\mathrm{of}\;(\mathrm{invaded}\;\mathrm{structure}\;\mathrm{of}\;\mathrm{GTVnx}\_\mathrm{OS}\;\cap \;\mathrm{invaded}\;\mathrm{structure}\;\mathrm{of}\;\mathrm{GTVnx}\_\mathrm{ref})}{\mathrm{Number}\;\mathrm{of}\;\mathrm{invaded}\;\mathrm{structure}\;\mathrm{of}\;\mathrm{GTVnx}\_\mathrm{ref}}\times 100\text{\%}$$ (4)

Where invaded anatomical structure was defined as its partial or complete inclusion within the GTVnx_OS or GTVnx_ref, with final confirmation reached by the observer or expert.

### 2.7 Consistency of CTVp

The consistency of CTVp between observers was quantified using DSC, Hausdorff Distance (HD) and Intraclass Correlation Coefficient (ICC), which were widely applied for similarity evaluation of target volume segmentation (12).

The DSC is defined as twice the size of the intersection divided by the sum of the 2 volumes and calculated with following Formula 5. DSC ranges from 0 (no overlap) to 1 (perfect overlap), with higher values denoting better agreement.

$$\mathrm{DSC}=2\times \frac{A\cap B}{A+B}\;(A\;\mathrm{and}\;B:\;\mathrm{two}\;\mathrm{volumes}\;\;\mathrm{of}\;\mathrm{segmentation}\;A\;\mathrm{and}\;B)$$ (5)

The HD was defined as the maximum shortest distance from any point in one volume to the other. To minimize the influence of outliers, the 95th percentile value (HD95) was used. Contrary to DSC, a lower HD95 value indicates higher geometric similarity.

Overall agreement among the volume of three oncologists' segmentation was assessed using a two-way random-effects model of ICC for absolute agreement with single-measure reliability (13).

The consistency of original CTVp and adjusted CTVp were evaluated and compared to investigate the impacts of GTVnx segmentation accuracy on CTVp consistency.

### 2.8 Dosimetric and biological impacts of GTVnx segmentation accuracy

For each patient, three VMAT plans were created based on target volumes of three OS. The minimum dose covering 95% (D95) of the PTVnx_ref (Planning target volume of reference GTVnx) was compared across three plans.

The TCP was estimated using Niemierko's equivalent uniform dose (EUD)-based model (Formula 6) (14, 15).

$$\mathrm{TCP}=\;\frac{1}{1+{\left(\frac{\mathrm{TC}D_{50}}{\mathrm{EUD}}\right)}^{4\gamma 50}}$$ (6)

Where TCD_50_ and *γ*50 were set as 61.59 Gy and 3.38 according to previous studies (15, 16).

### 2.9 Statistical analysis and plot

Normality was assessed using the Anderson–Darling test. Depending on distribution, the Friedman test was used for multi-group comparisons, followed by paired *t*-tests or Wilcoxon signed-rank tests for pairwise analysis. Pearson or Spearman correlation tests were applied as appropriate. A *p*-value <0.05 indicated statistical significance.

Volumetric metrics of DSC, HD95 and target volume were computed with the Radiotherapy module of 3D Slicer (version 5.8.1). Statistical analysis and visualization were performed in R Studio (version 2021.9.1.372) using the tidyverse and DVHmetrics packages, in which the EUD was computed using the getEUD() function with EUDa = −10 to emphasize low-dose regions affecting TCP (17).

## 3 Results

**Figure 3:**
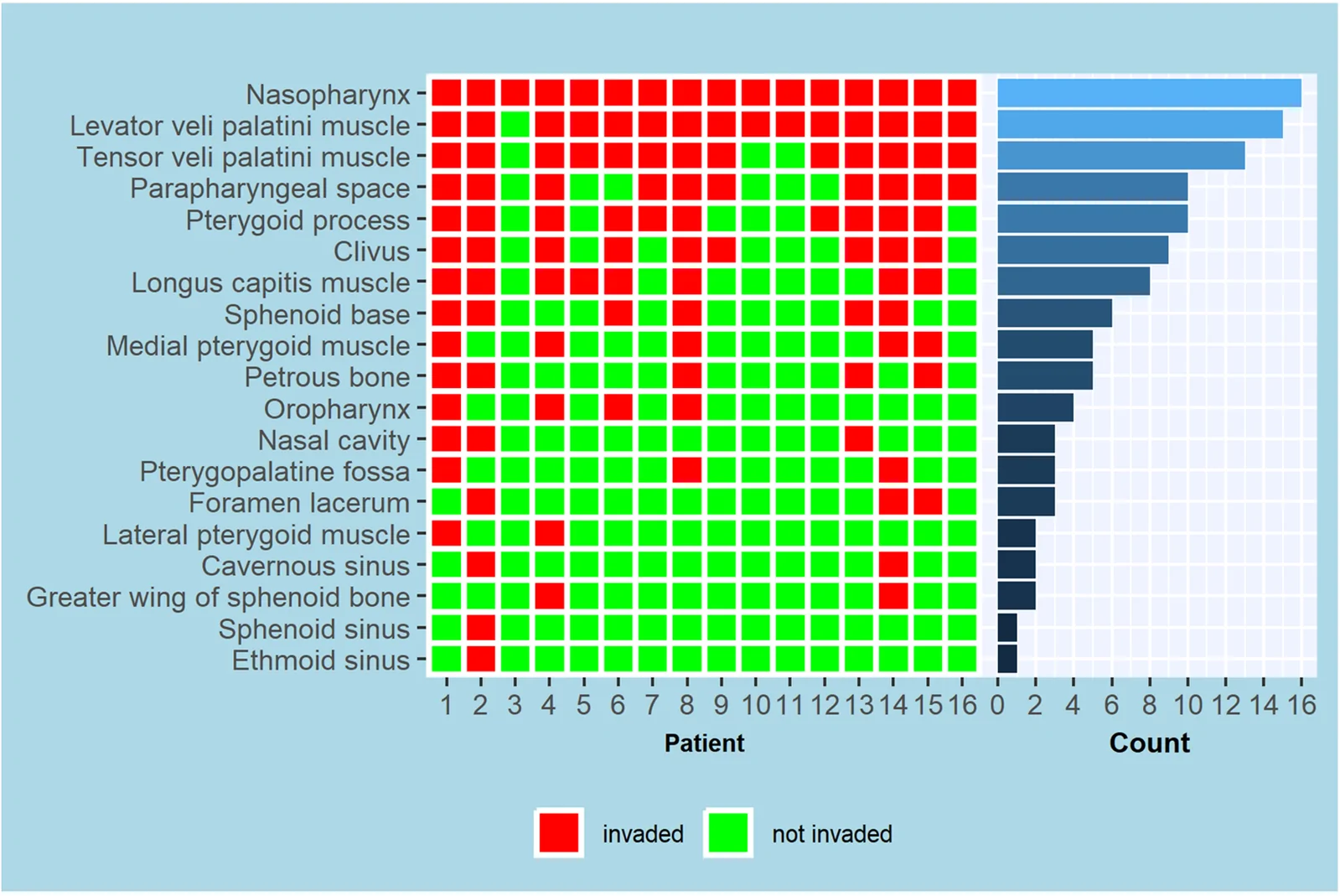
Statistics of invaded anatomical structures by nasopharyngeal tumor.

The complete list of evaluated anatomical structures and invaded anatomical structures by the nasopharyngeal tumor of each patient are presented in Figure 3.

**Figure 4:**
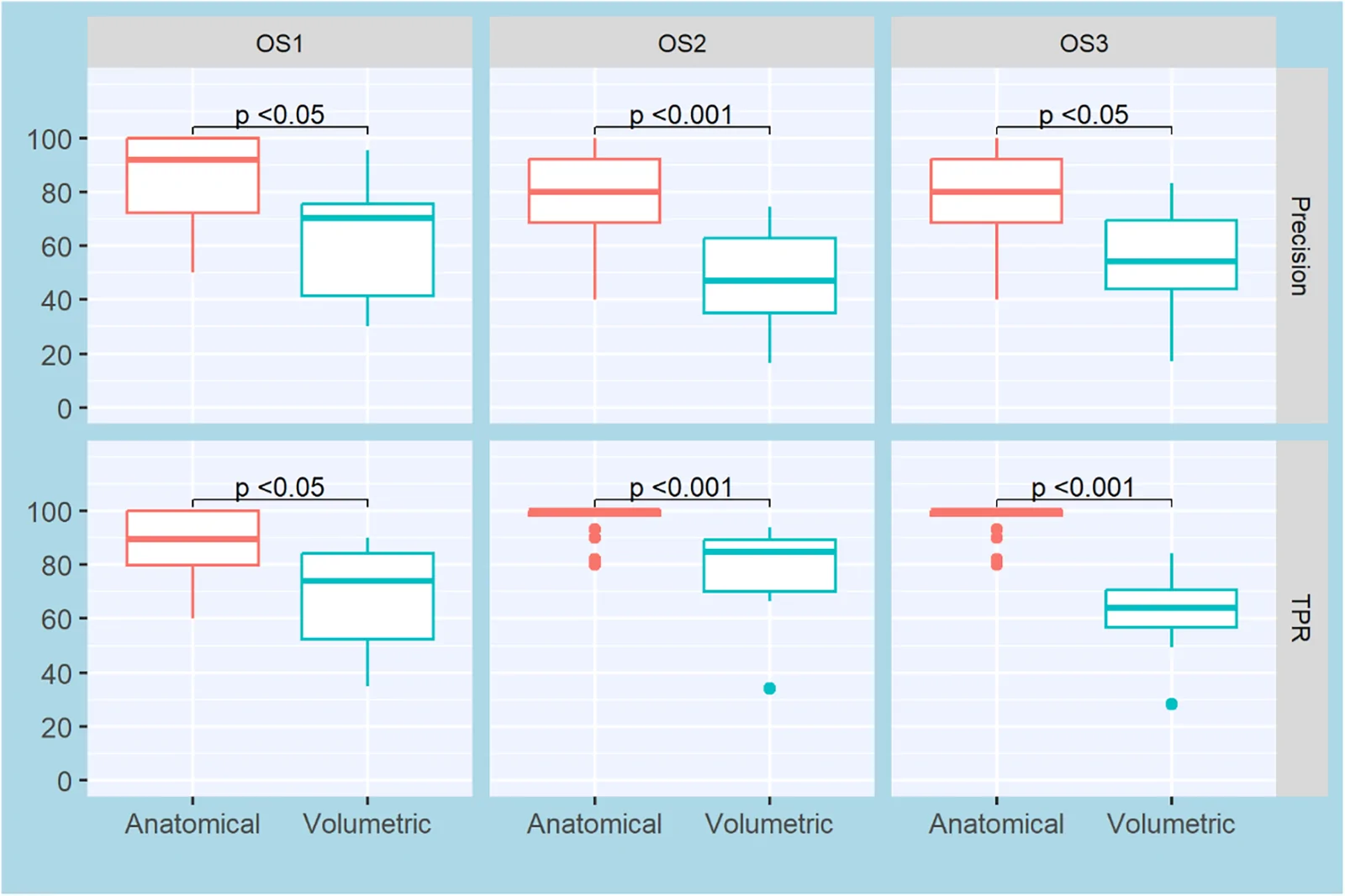
Anatomical and volumetric precision and true positive rate of GTVnx segmentation. The precision and TPR between anatomical and volumetric accuracy were compared with Wilcoxon paired test. TPR, True positive rate; OS1/2/3, Segmentation of observer 1/2/3.

The median volumetric precision and TPR of the three OS were 70.29%, 47.08%, 54.11% and 73.92%, 84.69%, 63.88%, which were significantly lower than anatomical values of 92%, 80%, 80% and 89.5%, 100%, 100%. (Figure 4, Supplementary Table 3).

Treatment plans based on three OS resulted in significantly lower D95 and EUD of reference PTVnx, leading to decreased TCP of 15.36%, 11.25%, and 24.77% on average, respectively (Table 1).

**Table 1:** Statistics and comparison of D95, equivalent uniform dose and tumor control probability of reference PTVnx.

Parameters	Observer segmentation	mean ± SD and *p*-value of Wilcoxon test				Δ mean ± SD	*p*-value***
		PTVnx of OS	*p*-value*	PTVnx of Reference	*p*-value**		
D95 (Gy)	OS1	70.23 ± 0.68	<0.001	62.40 ± 7.25	<0.001	−7.84 ± 7.04	<0.001
	OS2	69.93 ± 0.60		64.54 ± 6.00		−5.39 ± 5.8	<0.001
	OS3	70.12 ± 0.49		61.21 ± 6.00		−8.9 ± 5.93	<0.001
	Total	70.09 ± 0.60		62.72 ± 6.46		−7.38 ± 6.32	
EUD (Gy)	OS1	71.51 ± 0.39	0.65	66.94 ± 6.43	0.01	−4.57 ± 6.45	<0.001
	OS2	71.51 ± 0.51		68.35 ± 4.85		−3.16 ± 5.03	<0.001
	OS3	71.45 ± 0.47		63.46 ± 11.30		−7.99 ± 11.56	<0.001
		71.49 ± 0.45		66.25 ± 8.11		−5.24 ± 8.26	
TCP (%)	OS1	88.25 ± 0.77	0.64	72.88 ± 24.02	0.01	−15.36 ± 24.08	<0.001
	OS2	88.24 ± 0.97		77.00 ± 20.77		−11.25 ± 21.11	<0.001
	OS3	88.21 ± 0.87		63.36 ± 32.36		−24.77 ± 32.79	<0.001
	Total	88.21 ± 0.87		71.08 ± 26.25		−17.13 ± 26.52	

D95, minimum dose that covers 95% target volume; TCP, tumor control probability; EUD, equivalent uniform dose; PTVnx, planning target volume of pharyngeal tumor; OS1/2/3, observer segmentation; Δ, value of reference PTVnx—OS.

*/**Friedman test among TCP of 3 OS/Reference.

***Wilcoxon paired test between PTVnx of OS and Reference.

**Figure 5:**
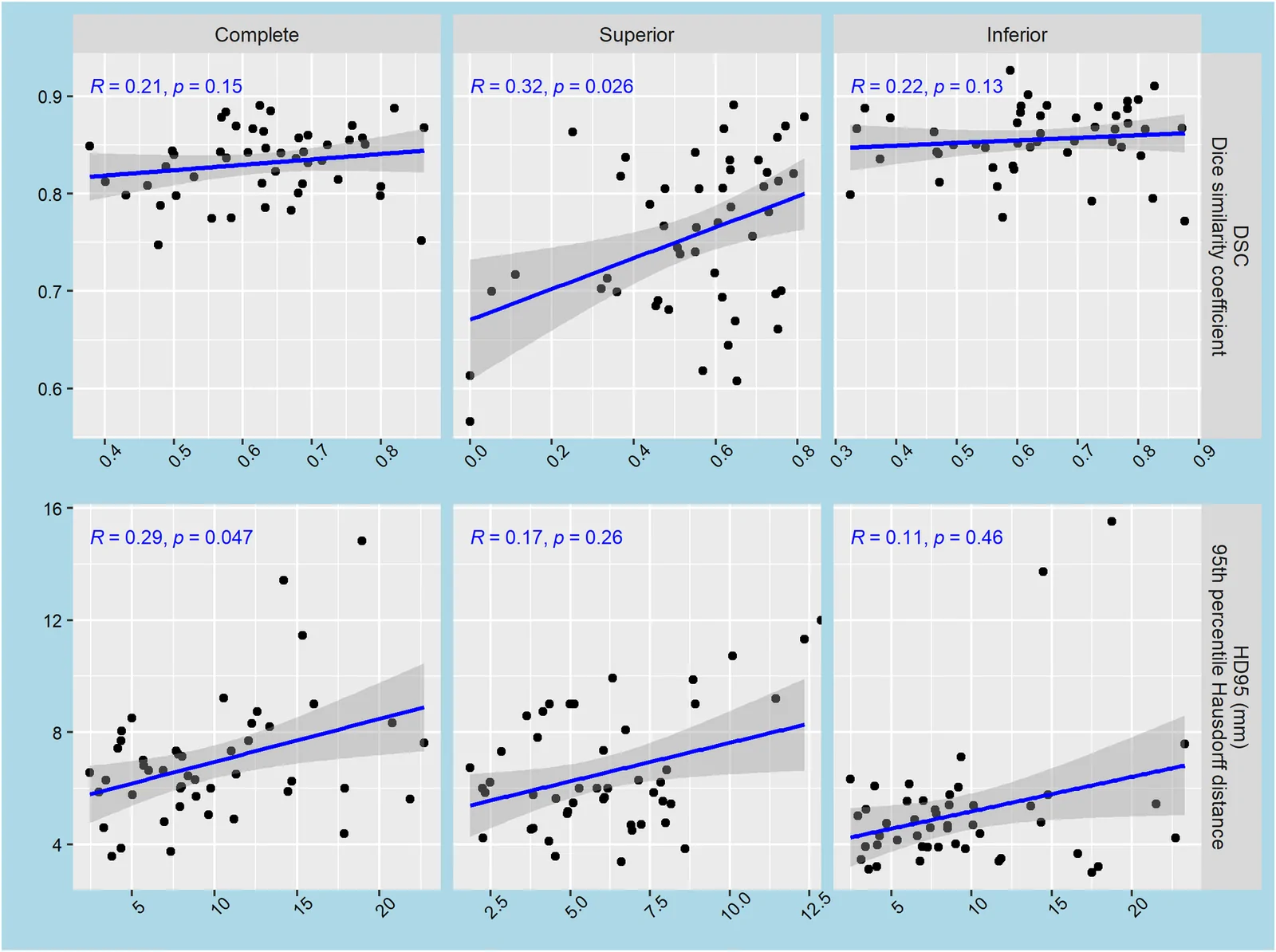
Spearman correlation test between similarity metrics of GTVnx and CTVp. *X*-axis: Similarity metrics of gross tumor volume (GTVnx); *Y*-axis: Similarity metrics of prophylactic clinical target volume of primary tumor (CTVp). The column labels (Complete, Superior, Inferior) correspond to the whole target volume and its subdivisions. The complete GTVnx/CTVp was divided into cranial (Superior) and caudal (Inferior) section by transverse plane at sphenoid sinus bottom on CT images.

**Figure 6:**
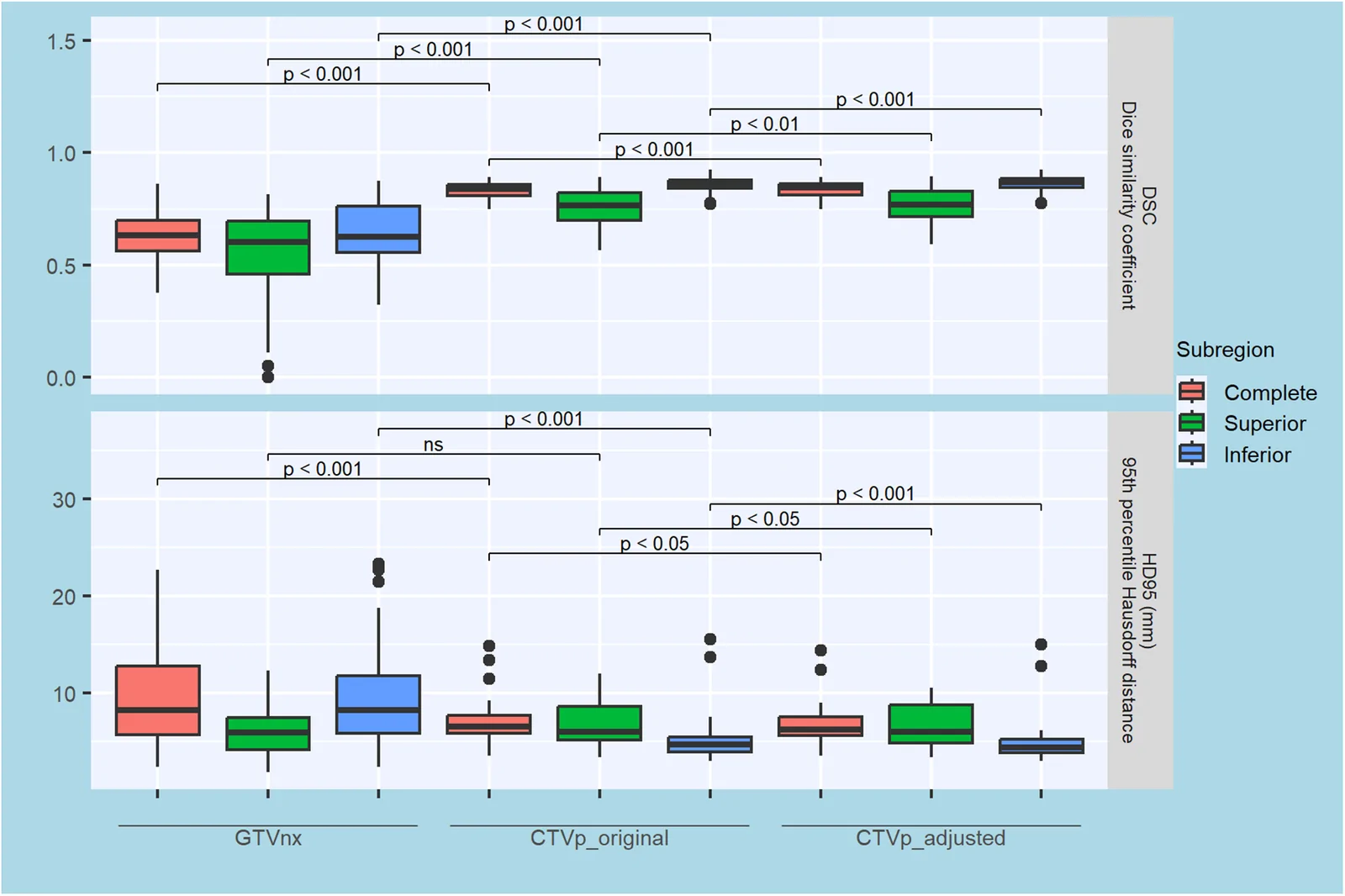
Boxplot and comparison of DSC and HD95 for GTVnx and CTVp between observer segmentations. Wilcoxon paired test was applied for comparisons between GTVnx and CTVp. The complete GTVnx/CTVp was divided into cranial (Superior) and caudal (Inferior) section by transverse plane at sphenoid sinus bottom on CT images, which were indicated with color red, green and blue.

Across all 3 OS, both CTVp_original and CTVp_adjusted showed significantly higher similarity than GTVnx except HD95 of superior CTVp, the median DSC and HD95 were 0.84 and 6.53 mm, 0.85 and 6.27 mm, 0.63 and 8.21 mm for complete original CTVp, adjusted CTVp and GTVnx, respectively. Significant differences were observed between superior and inferior section for both GTVnx and CTVp. Correlation analysis showed weak or non-significant associations between the similarity of GTVnx and CTVp. Differences of DSC and HD95 between the original and adjusted CTVp were minimal but statistically significant (Supplementary Table 4, Figures 5, 6).

The intra-class correlation coefficient (ICC) values of the inferior GTVnx, original CTVp, and adjusted CTVp were 0.96, 0.99, and 0.99, respectively—substantially higher than those of the superior section (0.60, 0.55, and 0.64). Notably, the ICC of the superior section increased slightly after CTVp adjustment.

Volume analysis showed all three observers overestimated the volume of GTVnx. Significant inter-observer difference was found in both superior and inferior GTVnx volumes while the difference across the CTVp of three OS were only observed in superior section (Table 2).

**Table 2:** Intra-class correlation coefficient for GTVnx, original and adjusted CTVp.

ROI	Section	Relative volume (mean ± SD)^a^			*p*-value*	ICC of absolute volume	
		OS1	OS2	OS3		ICC	95 CI
GTVnx	Complete	1.28 ± 0.61	2.10 ± 1.31	1.29 ± 0.65	<0.001	0.92	0.78–0.97
	Superior	1.36 ± 0.91	1.89 ± 0.91	0.88 ± 0.62	<0.001	0.60	0.29–0.82
	Inferior	1.62 ± 1.48	3.54 ± 4.74	2.07 ± 2.62	<0.001	0.96	0.87–0.99
Original CTVp	Complete	0.97 ± 0.06	1.03 ± 0.05	1.00 ± 0.06	0.05	0.98	0.96–0.99
	Superior	0.90 ± 0.14	1.20 ± 0.12	0.90 ± 0.16	<0.001	0.55	0.18–0.80
	Inferior	0.99 ± 0.05	0.97 ± 0.07	1.03 ± 0.06	0.57	0.99	0.98–1.00
Adjusted CTVp	Complete	0.98 ± 0.05	1.02 ± 0.05	1.00 ± 0.06	0.19	0.99	0.98–1.00
	Superior	0.91 ± 0.12	1.19 ± 0.12	0.90 ± 0.14	<0.001	0.64	0.22–0.86
	Inferior	1.00 ± 0.05	0.96 ± 0.07	1.03 ± 0.06	0.31	0.99	0.98–1.00

OS, observer segmentation; GTVnx, gross tumor volume of nasopharynx; Original CTVp, original segmentation of prophylactic clinical target volume of OS; Adjusted CTVp, adjusted CTVp according to reference GTVnx.

a Relative volume of GTVnx (CTVp) = volume of OS/volume of reference GTVnx (mean volume of 3 OS).

* *P*-value of Friedman test among the absolute volume of 3 OS.

## 4 Discussion

This study provides the first comprehensive evaluation of GTVnx segmentation accuracy in nasopharyngeal carcinoma radiotherapy at both volumetric and anatomical dimensions and their consequent dosimetric and biological impacts. Our findings reveal substantially low volumetric accuracy alongside notably high anatomical accuracy. The volumetric inaccuracies translated into clinically meaningful reductions in D95, EUD, and TCP for the reference PTVnx, with mean decreases of 7.38 Gy, 5.24 Gy, and 17.13%, respectively. Inter-observer consistency for CTVp segmentation markedly exceeded that for GTVnx, whether assessed on original or adjusted CTVp. Specifically, the median DSC/HD95 values were 0.63/8.21 mm for GTVnx, 0.84/6.53 mm for original CTVp, and 0.85/6.27 mm for adjusted CTVp. Under current consensus guideline, the statistically significant—though numerically modest—improvement of CTVp consistency across observers after adjustment based on reference GTVnx suggests that accurate GTVnx segmentation may further improve CTVp consistency.

Since the international consensus guidelines for segmentation of CTVp were recommended based on the invaded anatomical structures, evaluating GTVnx segmentation accuracy at both volumetric and structural levels is essential for understanding its downstream impact on CTVp consistency. To the best of our knowledge, this is the first study to explicitly assess segmentation accuracy at the anatomical structural level. Our findings suggest that, despite relatively low volumetric accuracy, the high anatomical accuracy may explain the observed robustness and consistency of CTVp segmentation across observers within the framework of the guideline.

Previous studies using pathological specimen reconstruction reported precision of 72%–89% for segmentation of laryngeal cancer with tending to overestimate tumor volume (18), while median sensitivity of 93% and median positive predictive value of 50% for hypopharyngeal tumor (19). For NPC, where pathological reconstruction is infeasible, the Simultaneous Truth and Performance Level Estimation (STAPLE), a consensus segmentation among multiple OS (20), has been widely used to establish ground truth for both GTV and CTV in head and neck cancers. Cardoso et al. (21) reported an average DSC of 0.72 and 0.76 for GTV and CTV among four oncologists' segmentations of ten head and neck cancer patients. Cardenas et al. (22) reported an average DSC of 0.77 across 26 oncologists for both GTV and CTV of four oropharyngeal cancer patients, which were both 0.67 compared with STAPLE. The HD95 across oncologists ranged from 5.2–11.9 mm for GTV and 9.4–15.7 mm for CTV, which were 3.7–8.9 mm and 7.3–11.4 mm compared to STAPLE.

Notably, abovementioned studies showed a comparable or even higher similarity of GTV than CTV, contradictory to our study. Several reasons may explain this discrepancy. First, NPC-specific guideline for CTV segmentation was adopted in our study. Training and establishing standardized guidelines have been proven capable of improving CTV segmentation consistency (3). Second, the difference in number of observers. Previous study has demonstrated the number of observers is a potential source of bias affecting the results in evaluating segmentation accuracy (23). Third, the anatomical complexity surrounding primary disease differs between sites.

The DSC, HD95, and ICC results collectively indicated higher geometric similarity among observers in the inferior sections of both original and adjusted CTVp than in the superior sections. This may be attributed to the complex anatomy near the skull base within the superior CTVp. Interestingly, both the value of DSC and HD95 for the superior GTVnx were lower than those of the inferior section. This apparent contradiction can be explained by the combined effect of metric definitions and target volume characteristics. The superior GTVnx volumes were significantly smaller and exhibited greater variation among observers. In such scenarios, even small distances between segmentations leading to a lower HD95 can result in a disproportionately large reduction in the relative overlap of the volumes leading to a lower DSC, as DSC is highly sensitive to the volumetric size of the structures being compared (24).

Only a limited number of studies have evaluated the dosimetric and TCP impacts of segmentation inaccuracy in NPC. Peng et al. (25) compared the GTV segmentation of 10 junior radiation oncologists to the STAPLE segmentation in a single NPC patient and reported a minimum prescription dose coverage of 77.99% for PTVnx of STAPLE, but TCP was not analyzed in their study. Chen et al. (26) evaluated target volume segmentation by four radiation oncologists in 12 NPC patients and observed reductions in prescription dose coverage ranging from 7.62% to 10.54%, resulting in average TCP decreases exceeding 1%, which is considerably smaller than the reductions observed in our study. Together with our findings, these studies underscore the substantial dosimetric and biological consequences of inaccurate GTVnx segmentation. Variations in reported TCP reductions may be attributable to differences in planning techniques and TCP modeling parameters.

The current study provides evidence that single-observer GTVnx segmentation is associated with notable volumetric inaccuracy and potentially compromising dose coverage and TCP, highlighting the necessity to improve GTVnx segmentation accuracy in routine clinical practice. Peer review process, radiologist involvement and multimodality imaging represent promising strategies to mitigate such inaccuracy and improve clinical outcomes (27, 28).

Several limitations should be acknowledged. First, using segmentation from a single senior radiologist as the reference may not be sufficiently robust for defining the true tumor volume, and could therefore introduce bias into the calculation of underdosage and TCP reduction. Nonetheless, the differences of estimated TCP between OS-based plannings still reflect the potential impacts of GTVnx segmentation inaccuracy on TCP due to the uniqueness of ground truth. Second, this study was not designed to directly evaluate the effectiveness of consensus guideline on CTVp segmentation consistency, but was conducted within its framework. The high anatomical accuracy of GTVnx segmentation, together with the high CTVp consistency, leads us to speculate that the former may have contributed to the latter—given that CTVp segmentation per the guideline is inherently anchored to invaded anatomical structures. This hypothesis warrants further confirmation. Third, current study included only 16 NPC patients, which may limit statistical power. However, this sample size remains relatively large compared to previous NPC-specific studies. Finally, due to the limited cohort size, the influence of specific anatomical invasion patterns on segmentation accuracy was not analyzed, which remains an important topic for future research.

## 5 Conclusions

In nasopharyngeal carcinoma, GTVnx segmentation inaccuracies are predominantly volumetric rather than anatomical. Such volumetric inaccuracy may potentially compromise target volume coverage and tumor control. Within the framework of consensus guidelines, the high consistency observed in CTVp segmentation may be attributable to the high anatomical accuracy of GTVnx segmentation—a hypothesis that warrants further investigation. Collectively, these findings underscore the need for future studies focused on improving volumetric accuracy in GTVnx segmentation.

## Data Availability

The original contributions presented in the study are included in the article/Supplementary Material, further inquiries can be directed to the corresponding author.
