# Supplementary material for: Impacts of volumetric vs. anatomical accuracies in GTV segmentation for nasopharyngeal carcinoma: a consensus-guideline-based study
Source: Front Med (Lausanne). 2026 Sep 8;13:1941973. doi: 10.3389/fmed.2026.1941973 (PMC13593484; doi:10.3389/fmed.2026.1941973)
Supplement: Supplementary file 1 [file Table1.docx]

Supplementary Table 1. Summarizes the target volume segmentation and prescribed doses.

| Target Volume | Definition | planning target volume: prescription dose |
| --- | --- | --- |
| GTVnx | Primary nasopharyngeal tumor(After induction chemotherapy: soft tissue lesions + prior bony lesions) | PTVnx: D95:70Gy/33Fractions |
| GTVln | Retropharyngeal lymph nodes with short-axis ≥ 5 mm;  cervical lymph nodes with short-axis ≥ 1 cm; showing central necrosis or ring enhancement (regardless of size);  ≥3 confluent nodes in high-risk areas | PTVln: D95:70Gy/33Fractions |
| CTV | Prophylactic area of primary tumor and lymph nodes | PTV:  D95: 54Gy/33Fractions |
