# Supplementary material for: Impacts of volumetric vs. anatomical accuracies in GTV segmentation for nasopharyngeal carcinoma: a consensus-guideline-based study
Source: Front Med (Lausanne). 2026 Sep 8;13:1941973. doi: 10.3389/fmed.2026.1941973 (PMC13593484; doi:10.3389/fmed.2026.1941973)
Supplement: Supplementary file 2 [file Table2.docx]

| Organs | Dose parameter | Constraint |
| --- | --- | --- |
| Brainstem PRV | D_max_ | <60Gy |
| Brainstem PRV | V60 | <1cc |
| Brain Stem | D_max_ | <54Gy |
| Larynx | D_mean_ | <36Gy |
| Left Lens | D_max_ | <6Gy |
| Right Lens | D_max_ | <6Gy |
| Optic chiasma | D_max_ | <60Gy |
| Left optic nerve | D_max_ | <60Gy |
| Right optic nerve | D_max_ | <60Gy |
| Oral cavity | D_mean_ | <36Gy |
| Left parotid | D_mean_ | <36Gy |
| Right parotid | D_mean_ | <36Gy |
| Pituitary | D_max_ | <60Gy |
| Spinal cord PRV | D_max_ | <45Gy |
| Spinal Cord | D_max_ | <40Gy |
| Thyroid | D_mean_ | <45Gy |
| D_mean_ : mean dose; D_max_ : Maximum dose; V70: The absolute volume received more than 60 Gy | | |

Supplementary Table 2. Dose constraints for different Organs.
