# Supplementary material for: Impacts of volumetric vs. anatomical accuracies in GTV segmentation for nasopharyngeal carcinoma: a consensus-guideline-based study
Source: Front Med (Lausanne). 2026 Sep 8;13:1941973. doi: 10.3389/fmed.2026.1941973 (PMC13593484; doi:10.3389/fmed.2026.1941973)
Supplement: Supplementary file 3 [file Table3.docx]

Supplementary Table 2. Anatomical and volumetric precision and true positive rate of GTVnx segmentation

| Level | Metrics | *  OS | Percentile value | | | | ****  *p*-value |
| --- | --- | --- | --- | --- | --- | --- | --- |
|  |  |  | 0 | 50 | 95 | 100 |  |
| Anatomical | Precision  (%) | OS 1 | 50.00 | 92.00 | 100.00 | 100.00 | 0.01 |
|  |  | OS 2 | 40.00 | 80.00 | 100.00 | 100.00 |  |
|  |  | OS 3 | 40.00 | 80.00 | 100.00 | 100.00 |  |
|  |  | Total | 40.00 | 80.50 | 100.00 | 100.00 |  |
|  | True positive rate(%) | OS 1 | 60.00 | 89.50 | 100.00 | 100.00 | <0.01 |
|  |  | OS 2 | 80.00 | 100.00 | 100.00 | 100.00 |  |
|  |  | OS 3 | 80.00 | 100.00 | 100.00 | 100.00 |  |
|  |  | Total | 60.00 | 100.00 | 100.00 | 100.00 |  |
| Volumetric | Precision  (%) | OS 1 | 30.15 | 70.29 | 87.82 | 95.39 | <0.01 |
|  |  | OS 2 | 16.60 | 47.08 | 72.08 | 74.52 |  |
|  |  | OS 3 | 17.23 | 54.11 | 80.62 | 83.33 |  |
|  |  | Total | 16.60 | 54.11 | 83.18 | 95.39 |  |
|  | True positive rate(%) | OS 1 | 34.94 | 73.92 | 89.40 | 90.04 | 0.01 |
|  |  | OS 2 | 34.02 | 84.69 | 93.13 | 93.94 |  |
|  |  | OS 3 | 28.31 | 63.88 | 80.78 | 84.27 |  |
|  |  | Total | 28.31 | 70.96 | 89.95 | 93.94 |  |
| *OS1/2/3: Segmentation of observer 1/2/3; **: *p*-value of Friedman test among 3 OS. | | | | | | | |
