# Supplementary material for: Impacts of volumetric vs. anatomical accuracies in GTV segmentation for nasopharyngeal carcinoma: a consensus-guideline-based study
Source: Front Med (Lausanne). 2026 Sep 8;13:1941973. doi: 10.3389/fmed.2026.1941973 (PMC13593484; doi:10.3389/fmed.2026.1941973)
Supplement: Supplementary file 4 [file Table4.docx]

Supplementary Table 3. The DSC and HD95 of GTVnx and CTVp for all three paired comparisons of observer segmentation.

| *Target volume | Similarity metrics | Section |  | Percentile value | | | | ****  *p*-value | *****  *p*-value |
| --- | --- | --- | --- | --- | --- | --- | --- | --- | --- |
|  |  |  |  | 0 | 50 | 95 | 100 |  |  |
| GTVnx | Dice similarity coefficient | Complete |  | 0.38 | 0.63 | 0.81 | 0.86 |  |  |
|  |  | Superior |  | 0 | 0.60 | 0.77 | 0.82 | - | - |
|  |  | Inferior |  | 0.32 | 0.63 | 0.83 | 0.88 |  |  |
|  | 95^th^ percentile Hausdorff distance (mm) | Complete |  | 2.44 | 8.21 | 20.14 | 22.71 |  |  |
|  |  | Superior |  | 1.85 | 5.93 | 9.79 | 12.34 | - | - |
|  |  | Inferior |  | 2.44 | 8.22 | 20.54 | 23.28 |  |  |
| CTVp_ Original | Dice similarity coefficient | Complete |  | 0.75 | 0.84 | 0.88 | 0.89 | <0.001 |  |
|  |  | Superior |  | 0.57 | 0.77 | 0.87 | 0.89 | <0.001 | - |
|  |  | Inferior |  | 0.77 | 0.86 | 0.90 | 0.93 | <0.001 |  |
|  | 95^th^ percentile Hausdorff distance (mm) | Complete |  | 3.57 | 6.53 | 10.68 | 14.83 | <0.001 |  |
|  |  | Superior |  | 3.38 | 6.00 | 11.11 | 12.00 | 0.43 | - |
|  |  | Inferior |  | 3.00 | 4.68 | 7.41 | 15.52 | <0.001 |  |
| CTVp_ Adjusted | Dice similarity coefficient | Complete |  | 0.75 | 0.85 | 0.89 | 0.89 | <0.001 | <0.001 |
|  |  | Superior |  | 0.59 | 0.77 | 0.87 | 0.89 | <0.001 | 0.001 |
|  |  | Inferior |  | 0.77 | 0.87 | 0.91 | 0.93 | <0.001 | <0.001 |
|  | 95^th^ percentile Hausdorff distance (mm) | Complete |  | 3.57 | 6.27 | 9.00 | 14.39 | <0.001 | 0.015 |
|  |  | Superior |  | 3.38 | 6.00 | 9.83 | 10.57 | 0.51 | 0.019 |
|  |  | Inferior |  | 3.00 | 4.44 | 6.17 | 15.02 | <0.001 | <0.001 |
| * GTVnx: Gross tumor volume of nasopharynx;  CTVp_original/adjusted: Original/adjusted prophylactic clinical target volume of primary tumor.  ** *p* value of Wilcox paired test between CTVp and GTVnx;  ****p* value of Wilcox paired test between CTVp_original and CTVp_adjusted | | | | | | | | | |
